# A cross-sectional examination of modifiable risk factors for chronic disease among a nationally representative sample of youth: are Canadian students graduating high school with a failing grade for health?

**DOI:** 10.1186/1471-2458-13-569

**Published:** 2013-06-11

**Authors:** Scott T Leatherdale, Vicki Rynard

**Affiliations:** 1 School of Public Health and Health Systems, University of Waterloo, 200 University Avenue, Waterloo ON, N2L 3G1, Canada; 2 Propel Centre for Population Health Impact, University of Waterloo, 200 University Avenue, Waterloo ON, N2L 3G1, Canada

**Keywords:** Obesity, Physical activity, Tobacco, Alcohol, Marijuana, Diet, Youth

## Abstract

**Background:**

Substance use and weight gain among youth increase the risk for future disease. As such, the purpose of this study is to examine how many Canadian youth are currently failing to meet substance use and weight gain related public health guidelines.

**Methods:**

Data from the 2010–11 Youth Smoking Survey were used to examine grade 9 to 12 students meeting seven different guidelines by sex and by grade.

**Results:**

Among Canadian youth, 8.8% were current smokers, 18.8% were current marijuana users, 25.5% were current binge drinkers, 22.5% were considered overweight or obese, 31.2% did not meet physical activity guidelines, 89.4% exceeded sedentary behaviour guidelines, and 93.6% reported inadequate fruit and vegetable intake. The mean number of risk factors per student was 2.9 (±1.2); only 0.5% of youth reported having none of the risk factors.

**Conclusion:**

Students rarely met all seven public health guideline examined, and the vast majority of actually reported having two or more modifiable risk factors for disease.

## Background

Age-related increases in substance use and excess weight-gain that occur among youth are cause for concern as they are associated with numerous negative health outcomes [1-9]. Substance use and obesity (and the correlates of obesity) tend to be established during adolescence [2,10-12], and most Canadian youth exhibit one or more of these risk factors for future morbidity [10-15]. For instance, according to data from grade 9 to 12 students in the 2008 Youth Smoking Survey (YSS), 8.9% were current smokers (increasing from 6.8% to 15.6% between grade 9 and 12), 18.8% were current marijuana users (increasing from 17.7% to 29.1% between grade 9 and 12), and 27.0% were current binge drinkers (increasing from 21.0% to 50.8% between grade 9 and 12) [10]. Data from the 2007–09 Canadian Health Measures Survey (CHMS) suggest that among Canadian youth aged 15 to 19, 31% of boys and 26% of girls are overweight or obese [12]. In terms of the correlates for obesity, the 2007–09 CHMS identified that only 9% of boys and 4% of girls accumulate 60 minutes of moderate-to-vigorous physical activity (PA) on at least 6 days a week [13], the 2008 YSS identified that the average daily sedentary screen time among Canadian youth was 7.8 (± 2.3) hours per day [11], and the 2011 Canadian Community Health Survey (CCHS) identified that only 43.7% of males and 44% of females aged 12 to 19 years consumed five servings of fruits and vegetables daily [14]. It is critical to promote healthier lifestyles among Canadian youth.

While the aforementioned modifiable risk factors for disease are individually important, evidence suggests that having more than one risk factor can act to amplify the risk [15]. Considering that it is common for youth to exhibit more than one of these risk factors [11,12,16-20], it is not only important to understand how many Canadian youth are currently failing to meet the Canadian public health guidelines associated with these risk factors [3,21-26], but it is also important to determine how many youth are at potentially increased risk due to co-occurrence of more than one risk factor. Although some evidence has previously been published on the co-occurrence of modifiable risk factors among youth in Canada using data from the National Longitudinal Survey of Children and Youth (NLSCY) [27-29], that evidence is subject to some major limitations for informing current prevention programming. For instance, although the most recent evidence presented by Alamian and Paradis [27] are based on longitudinal data, there are four limitations associated with that evidence which warrant new research in this domain: 1) the most recent wave of data examined were from 2004–2005, as such those data are no longer as informative for informing current prevention programming as data from 2010; 2) they examined ever use of tobacco and ever use of alcohol whereas current tobacco or current alcohol use may be more informative, 3) due to changes in guidelines, the measures for tobacco, alcohol use, sedentary behaviour and PA were not operationally defined according to definitions within the current prevention guidelines; and 4) the according to Statistics Canada [30], the NLSCY was only nationally representative to the original sample population (Cycle 1). Although these earlier reports are important [27-29], additional investigation into this issue is warranted with the most current nationally representative youth data available.

Considering schools provide an effective environment for intervening with youth [31], and international guidelines recommend a comprehensive approach to achieving health promoting schools [32,33], the objective of this study is to use nationally representative data collected from grade 9 to 12 students to determine the prevalence for the these major modifiable risk factors for disease and examine how prevalence rates and co-occurence of risk factors change across grades.

## Methods

### Design

This study used data collected from 31,396 students in grades 9 to 12 who responded to the substance use section of the 2010–11 Canadian Youth Smoking Survey (2010 YSS); a nationally representative school-based survey of youth in Canada. In brief, the population of interest for the data used in this study consisted of all young Canadian residents in grades 9 to 12 attending public and private secondary schools in nine Canadian provinces; youth residing in the Yukon, Nunavut, the Northwest Territories, and New Brunswick were excluded from the population of interest, as were youth living in institutions or on First Nation Reserves, and youth attending special schools or schools on military bases. While New Brunswick participated in all prior cycles of YSS, the provincial government chose not to participate in 2010/2011. The survey design and sample weights allow us to produce population-based weighted sample estimates within this manuscript. The University of Waterloo Office of Research Ethics and appropriate School Board and Public Health Ethics committees approved all procedures. Detailed information on the 2010 YSS sample design, methods, response rates and measures are available at http://www.yss.uwaterloo.ca/results/yss10_user_guide_english_ver5_20120411.pdf.

### Measures

The operational definitions for the measures used in this manuscript are consistent with previous research using national standards [10,11,34-36] or current national public health guidelines [22,23,25,26]. This allows us to be consistent with the standards set forth and used by the different Canadian public health authorities who are responsible for (a) determining what risk factor measures are health promoting or health inhibiting, and (b) developing and implementing population-level strategies for intervening. For instance, our measures of tobacco use, alcohol use, marijuana use, obesity, and fruit and vegetable intake are consistent with the surveillance measured used by Health Canada and the Public Health Agency of Canada (PHAC), and our measures of physical activity and sedentary behaviour are consistent with the guidelines developed by the Canadian Society for Exercise Physiology and used by Health Canada and PHAC.

#### ***Substance Use***

Tobacco use was assessed by asking respondents, “Have you ever smoked 100 or more whole cigarettes in your life?” and “On how many of the last 30 days did you smoke one or more cigarettes?”. Consistent with previously validated measures of current smoking [36], students who reported ever smoking 100 cigarettes and any smoking in the previous 30 days were classified as current smokers. Marijuana use was assessed by asking respondents, “In the last 12 months, how often did you use marijuana or cannabis? (a joint, pot, weed, hash…)”. Those who reported marijuana use once a month or more were classified as current marijuana users. Binge drinking (5 or more drinks on one occasion) alcohol use was assessed by asking respondents, “In the last 12 months, how often did you have 5 drinks of alcohol or more on one occasion?”. Those who reported binge drinking once a month or more were classified as current binge drinkers.

#### ***Overweight/Obesity and Correlates of Overweight/Obesity***

Using previously validated measures of self-reported height and weight [35], Body Mass Index (BMI) was calculated for each student using the measures of weight (kg) and height (m) (BMI = kg/m^2^). Weight status was then determined using the BMI classification system of the World Health Organization [26] based on age and sex adjusted BMI cut-points. Using previously validated measures [35], physical activity was measured by asking respondents how many minutes of hard PA they engaged in on each of the last seven days. Consistent with the Canadian PA guidelines for youth [22], respondents who did not report performing hard PA a day for at least three out of the last seven days were classified as being inactive. Respondents were asked to report the average number of hours per day that they spent (a) texting or talking on the phone, (b) e-mailing or instant messaging, (c) playing video games, (d) playing or surfing on a computer, or (e) watching TV or movies. Respondents could choose from “none,” “less than 1 hour a day,” “1 to 2 hours a day,” “more than 2 hours a day but less than 5 hours a day,” or “5 or more hours a day” for each category. We then calculated a conservative estimate of the mean sedentary behaviour time per day based on the sum of lowest values for each response category reported, except for the category “less than 1 hour per day” where 0.5 hours was used. Consistent with the Canadian sedentary behaviour guidelines for youth [23], respondents were classified as highly sedentary if they reported an average of more than two hours of sedentary behaviour a day. Respondents were asked to report how many servings of fruits and/or vegetables they eat on a usual day. Consistent with the Canada Food Guide fruit and vegetable consumption recommendations for teens [25], males who reported less than eight servings per day and females who reported less than seven servings per day were classified as having inadequate fruit and vegetable consumption^a^.

### Analyses

Descriptive analyses of the risk factors were examined by sex and by grade. As evident in Table 1, these data were not normally distributed. We also determined the total number of risk factors per student to examine the co-occurence of multiple risk factors by sex and by grade. In all analyses, survey weights were used to adjust for non-response between provinces and groups, thereby minimizing any bias in the analyses caused by differential response rates across regions or groups. For missing data, imputations were not performed; as such, the prevalence of each risk factor was based on the sample that had complete data for that particular indicator. This allowed us to preserve as much of the sample data as possible. Specific details on the amount of missing data for the measures in 2010–11 YSS have been previously published online [http://www.yss.uwaterloo.ca/_global/documents/yss10_frequencies_publicuse_english_ver3_20120125.pdf]. When examining the co-occurrence of multiple risk factors by grade and sex, respondents with missing data for any one or more indicators were excluded from the analyses. Significance was assessed using the chi-square test. The statistical package SAS 9.2 was used for all analyses [37].

**Table 1:** **Descriptive statistics for estimates of risk factor prevalence by sex for the population**, **2010**-**2011**, **Canada**

		**Male**	**Female**	**Total**
		**(n = 849,600) %**^**a**^	**(n = 791,400) %**^**a**^	**(n = 1,641,000) %**^**a**^
Grade	9	24.8	25.1	24.9
	10	26.1	25.4	25.8
	11	25.5	25.7	25.6
	12	23.6	23.8	23.7
Current Smoker *(based on cigarettes only)*	Yes	9.7	7.7	8.8
	No	90.3	92.3	91.2
Current Marijuana Use	Yes	22.3	15.3	18.8
	No	77.7	84.7	81.2
Current Binge Drinking	Yes	27.7	23.1	25.5
	No	72.3	76.9	74.5
Weight Status	Overweight/Obese	29.1	15.2	22.5
	Normal Weight	70.9	84.8	77.5
Physical Activity Level	Inactive	23.9	39.0	31.2
	Active	76.1	61.0	68.8
Sedentary Behaviour Level	High	89.8	89.1	89.4
	Moderate / Low	10.2	10.9	10.6
Fruit / Vegetable Consumption	Inadequate	94.5	92.7	93.6
	Adequate	5.5	7.3	6.4
		**Male**	**Female**	**Total**
		**(n = 577,600) %**^**b**^	**(n = 518,400) %**^**b**^	**(n = 1,096,000) %**^**b**^
Number of Risk Factors	All 7	0.3	#	0.3
	6	2.5	1.9	2.2
	5	7.4	5.9	6.7
	4	18.4	14.2	16.4
	3	32.7	35.8	34.2
	2	33.4	33.2	33.3
	1	5.1	7.9	6.4
	None	#	0.9	0.5

^a^ weighted population estimate where missing data are excluded from the estimate.

^b^ weighted population estimate where respondents were excluded if any individual risk factor data were missing.

^1^ Prince Edward Island, Nova Scotia, Newfoundland & Labrador.

^2^ Quebec does not include students in Grade 12.

^3^ Alberta, Saskatchewan, Manitoba.

# Data not reportable due to low cell size.

## Results

Table 1 presents estimates of risk factor prevalence by sex for the population. The population was 51.8% (n = 849,600) male and 48.2% (n = 791,400) female. Overall, 8.8% (n = 143,500) were current smokers, 18.8% (n = 297,200) were current marijuana users, and 25.5% (n = 408,800) were current binge drinkers. Males were more likely than females to be current smokers (p < 0.001), current marijuana users (p < 0.001), and current binge drinkers (p < 0.001). The mean BMI among males was 22.6 (±4.5) kg/m^2^ and 21.4 (±3.9) kg/m^2^ among females. We identified that 22.5% (n = 276,100) of Canadian youth were considered overweight or obese for their age and sex, with males being substantially more likely than females to be considered overweight or obese (p < 0.001). BMI data were missing from 24.0% of male students and 26.8% of female students. In total, 31.2% (n = 464,300) of students did not meet the PA guideline, 89.4% (n = 1,463,900) were considered as being highly sedentary, and 93.6% (n = 1,487,400) reported inadequate fruit and vegetable consumption. Females were more likely than males to be inactive (p < 0.001), whereas males were more likely than females to report inadequate fruit and vegetable consumption (p < 0.001); sedentary behaviour did not significantly vary by sex (p = 0.06). The mean number of co-morbid risk factors among respondents was 2.9 (±1.2). Among Canadian students in grades 9 to 12, only 0.5% (n = 6,000) reported having none of the risk factors and only 0.3% (n = 2,800) reported having all 7 risk factors. The mean number of co-morbid risk factors was higher among males [3.0 (±1.2)] than females [2.8 (±1.1)] (p < 0.001).

**Figure 1:**
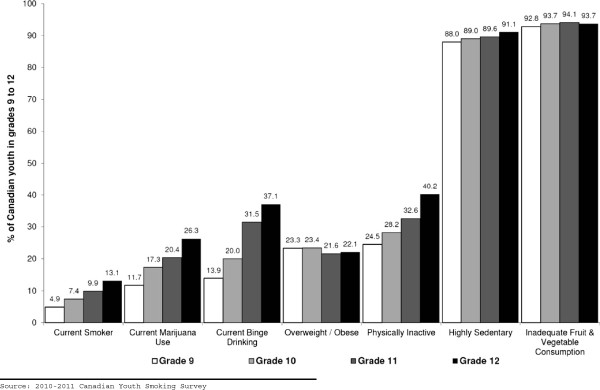
Prevalence of major modifiable risk factors for chronic disease among Canadian students by grade.

Figure 1 displays changes in the prevalence of risk factors by grade. As shown, there were consistent increases in the prevalence of current smoking, current marijuana use, current binge drinking, and physical inactivity among students across grades 9 to 12. The largest relative increases between grade 9 and 12 were observed for smoking (170%) and current binge drinking (167%). There was also a 124% relative increase in the prevalence of current marijuana use and a 64% relative increase in the prevalence physical inactivity across grade 9 to 12. There appears to be a ceiling effect for students being considered highly sedentary or having inadequate fruit and vegetable consumption, as there was only a 3% relative increase in the prevalence of being highly sedentary and a 1% relative increase in the prevalence of having inadequate fruit and vegetable consumption across grades 9 and 12. Overweight/obesity was the only risk factor where there was a relative decline (5%) in prevalence across grade 9 and grade 12.

**Figure 2:**
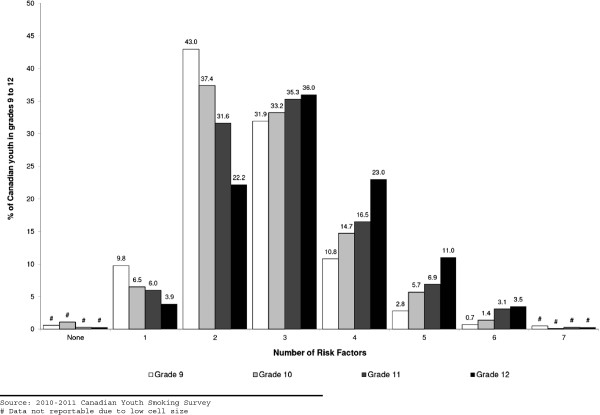
Prevalence of co-occurrence of modifiable risk factors for chronic disease among Canadian students by grade.

The mean number of co-morbid risk factors for students was 2.6 (±1.0) in grade 9, 2.8 (±1.1) in grade 10, 3.0 (±1.2) in grade 11, and 3.3 (±1.3) in grade 12. Figure 2 displays the changes in the prevalence of co-morbid risk factors by grade. Although there were no differences by grade for student reporting either no risk factors or all seven risk factors, it appears that the co-occurence of risk factors tends to be more common among students in higher grades. Interestingly, there also appears to be a transition point for the co-occurrence of three or more risk factors as a function of grade. For instance, there was a 61% relative decrease in the prevalence of respondents reporting only one risk factor and a 48% relative decrease in the prevalence of respondents reporting two risk factors across grade 9 to 12. Conversely, there was a 112% relative increase in the prevalence of respondents reporting four risk factors, a 296% relative increase in the prevalence of respondents reporting five risk factors, and a 432% relative increase in the prevalence of respondents reporting six risk factors across grade 9 to 12.

## Discussion

These data indicate that there is an urgent need to improve the health profile of Canadian secondary school students in order to avoid a large future burden of preventable disease. Not only was it rare to find students who meet all of the public health guideline indicators examined, but the vast majority of Canadian students actually reported having two or more modifiable risk factors for disease. This is concerning as the Centers for Disease Control and Prevention (CDC) report that the modifiable risk factors examined in this report represent the largest threats to future adult morbidity and mortality [38]. Considering that the prevalence estimates we present are consistent with U.S. estimates from the 2010–11 Youth Risk Behavior Surveillance System (YRBSS)[39] we are confident in these results.

Given the health and social effects associated with high risk substance use, and that substance abuse among adults is typically established during adolescence [10,39-41], the large number of Canadian youth we identified as being frequent substance users suggests that there is a need for ongoing and improved substance use prevention within Canadian secondary schools. The substantial variation in prevalence rates we identified across grades is consistent with previous research [10] and highlights the importance of targeting prevention interventions to different grades within a school. This also demonstrates why surveillance reports among youth that only present overall estimates may be misleading. There are often large differences between the prevalence rates when a student first starts secondary school (grade 9) to when they are preparing to leave secondary school (grade 12). Furthermore, considering that evidence has previously demonstrated that poly-substance use is common among Canadian youth [27,41], and our data suggest that the rates of co-morbid risk factors increase by grade, these results suggest that the current approach in school-based prevention programming, where most school-based programs do not focus on poly-substance prevention [42,43], are likely flawed. There is clearly a need to test and robustly evaluate the impact of poly-substance use prevention programs, especially if targeted to the students in higher grades who appear to be at the greatest risk [10].

There is a complex inter-relationship between overweight and obesity with sedentary behaviours, physical inactivity, and diet. There is emerging interest in examining the impact sedentary lifestyles are having on youth health [44], as youth obesity trends coincide with the prevalence of youth reporting more than four hours of screen time per day [45]. Although our results suggest that over one in five Canadian secondary school students is overweight or obese, prevalence rates of overweight and obesity remained relatively stable across grades. This was surprizing considering our data show that rates of physical inactivity increase dramatically across grades, but it may also be a function of missing BMI data. Even more alarming was our finding that rates of inadequate fruit and vegetable consumption and sedentary behaviour were so high that there was little room for variation across grades due to the ceiling effect observed. These two characteristics were the most common modifiable risk factors for future disease and represent the immediate priority areas for action. Considering the high prevalence of these behaviours, even among students in grade 9, it is clear that intervention efforts to reduce sedentary behaviour and to increase fruit and vegetable intake must begin prior to secondary school.

### Limitations

This study has several limitations common to survey research. Although the response rate was high and the data were weighted to help account for non-response, the findings are nevertheless subject to sample bias. In addition, the findings likely reflect some under-reporting for substance use and missing data for BMI as is common in survey research [46,47]. Although YSS data are based on self-reported measures, the questionnaire measures for current smoking, physical activity and BMI have previously demonstrated satisfactory reliability and validity [35,36]; the YSS measures for sedentary behaviour, alcohol use, marijuana use, and fruit and vegetable intake have not been previously validated. However, honest reporting was encouraged by ensuring confidentiality during data collection. It should also be noted that the cross-sectional nature of the design does not allow for causal inferences regarding trends over time. Longitudinal data are required to determine the temporal sequence of the onset of use for these substances and behaviours.

## Conclusions

In sum, the finding that large proportions of graduating students fail to meet many or most health behaviour guidelines should give all stakeholders cause for concern. It is clear that we need effective models of intervention. In order to reduce the impending burden associated with current rates of these modifiable risk factors for disease among youth populations in Canada, there is a need to re-orient prevention efforts aimed at modifying these individual behaviours into areas which offer the potential for population-wide impact [48]. A population-level intervention approach that changes the environment surrounding youth may have greater potential to affect long-term population-level reductions in youth risk behaviours [49,50]. Research has shown that population impact is best understood using ecological theory, a perspective that views behaviour as involving relationships between the individual and the multiple contexts in which they are situated [51,52]. Among youth populations, a school represents an ecological environment that can influence risk behaviours since youth spend ~25 hours each week in school throughout the school year where they could be influenced by people, programs, policies or the built environment (resources) to promote healthier lifestyles. Moreover, in order to optimize limited prevention resources, it may be even more beneficial if such population-level intervention efforts targeted multiple modifiable behavioural risk factors simultaneously. Considering the lack of current longitudinal research focusing on multiple risk behaviours in Canada, there is an immediate need for ongoing surveillance, research and evaluation on youth risk behaviours and the school-level characteristics (programs, policies, resources) associated with those behaviours which are amenable to modification.

## Endnote

^a^As a result of some wording differences for measuring fruit and vegetable intake between the English and French language questionnaires used in the 2010 YSS (as outlined in the 2010 YSS user guide), we could not differentiate between French language female respondents who had 6 or 7 servings of fruit or vegetables per day. As such, for these French language female respondents (16.7% of all females), we decided to use a more conservative estimate of fruit and vegetable intake based on the data available, where the French language female respondents were deemed as having inadequate fruit and vegetable intake if they consumed less than six servings per day.

## Abbreviations

BMI: Body mass index; CDC: Centers for disease control and prevention; CHMS: Canadian health measures survey; NLSCY: National longitudinal survey of children and youth; PA: Physical activity; PHAC: Public Health Agency of Canada; YRBSS: Youth risk behavior Surveillance system; YSS: Youth smoking survey.

## Competing interests

Both authors declare that they have no competing interests.

## Authors’ contributions

SL conceived the purpose, design and analyses for the study, led the interpretation of data, led the writing of the manuscript, developed the table and figures, and gave final approval of the version to be published. VR performed the analyses, reviewed the drafted the article and provided critical input, and gave final approval of the version to be published.

## Pre-publication history

The pre-publication history for this paper can be accessed here:

http://www.biomedcentral.com/1471-2458/13/569/prepub
